# Chemical Changes of Hydroperoxy-, Epoxy-, Keto- and Hydroxy-Model Lipids under Simulated Gastric Conditions

**DOI:** 10.3390/foods10092035

**Published:** 2021-08-29

**Authors:** Gloria Márquez-Ruiz, Francisca Holgado, María Victoria Ruiz-Méndez, Joaquín Velasco

**Affiliations:** 1 Instituto de Ciencia y Tecnología de Alimentos y Nutrición, Consejo Superior de Investigaciones Científicas (ICTAN-CSIC), 28040 Madrid, Spain; f.holgado@csic.es; 2 Instituto de la Grasa, Consejo Superior de Investigaciones Científicas (IG-CSIC), 41089 Sevilla, Spain; mvruiz@ig.csic.es (M.V.R.-M.); jvelasco@ig.csic.es (J.V.)

**Keywords:** epoxystearate, hydroperoxides, hydroxystearate, in vitro gastric digestion, methyl linoleate, lipid oxidation compounds, oxostearate

## Abstract

Chemical changes occurring in dietary lipid oxidation compounds throughout the gastrointestinal tract are practically unknown. The first site for potential chemical modifications is the stomach due to the strong acidic conditions. In this study, model lipids representative of the most abundant groups of dietary oxidation compounds were subjected to in vitro gastric conditions. Thus, methyl linoleate hydroperoxides were used as representative of the major oxidation compounds formed in food storage at low and moderate temperatures. Methyl 9,10-epoxystearate, 12-oxostearate and 12-hydroxystearate were selected as model compounds bearing the oxygenated functional groups predominantly found in oxidation compounds formed at the high temperatures of frying. Analyses were performed using gas-liquid chromatography/flame ionization detection/mass spectrometry and high performance-liquid chromatography/ultraviolet detection. Losses of methyl 9,10-epoxystearate and linoleate hydroperoxides in the ranges 17.8–58.8% and 42.3–61.7% were found, respectively, whereas methyl 12-oxostearate and methyl 12-hydroxystearate remained unaltered. Although quantitative data of the compounds formed after digestion were not obtained, methyl 9,10-dihydroxystearate was detected after digestion of methyl 9,10-epoxystearate, and some major volatiles were detected after digestion of linoleate hydroperoxides. Overall, the results showed that significant modifications of dietary oxidized lipids occurred during gastric digestion and supported that the low pH of the gastric fluid played an important role.

## 1. Introduction

Lipid oxidation products have attracted much attention because of the wide variety of degenerative processes and diseases associated, including cardiovascular disease, cancer and chronic inflammatory diseases [1,2,3]. However, still practically unknown is the contribution of lipid oxidation compounds coming from the diet and those formed endogenously. In the last few years, a number of reports have shown that lipid oxidation can occur under gastrointestinal conditions. In this regard, Kanner and coworkers described for the first time the stomach as a “bioreactor” in which oxidation and antioxidation reactions take place [4]. They carried out interesting in vitro experiments using simulated or human gastric fluids [1,5,6,7,8]. As to the duodenal phase of the digestive process, the analysis of lipolysis products formed in vitro was considerably improved by the development and application of liquid chromatography-light scattering detector-mass spectrometry and fast ultrahigh performance liquid chromatography-electrospray ionization-mass spectrometric methods [9,10]. In other studies, using an in vitro static gastrointestinal digestion model, and proton nuclear magnetic resonance spectroscopy and solid phase microextraction-gas chromatography/mass spectrometry for analyses, several kinds of primary and secondary oxidation were reported to be formed from digested oils [11,12,13,14]. By applying the same procedures, the influence of antioxidants and other minor components on oil oxidation during in vitro gastrointestinal digestion was additionally studied [15,16,17,18]. Dynamic gastrointestinal models have been also applied, specifically to determine the levels of short-chain aldehydes formed during digestion of marine oils [19,20,21]. The results obtained so far regarding the oxidation of lipids under digestive conditions, using different models, digestive phases and analytical methodologies, have been recently discussed in a comprehensive review [22].

However, the chemical changes occurring to dietary oxidation lipid compounds before absorption, which is the topic of the present research, are practically unknown. To the best of our knowledge, only Guillén and coworkers have shown that further oxidation reactions occurred in oxidized oils after in vitro gastrointestinal conditions and reported that some of the hydroperoxides originally present in oxidized sunflower oil were reduced to hydroxyl derivatives [23,24,25].

For oxidized lipid compounds, the first site for potential chemical modifications is expected to be the stomach due to the strong acidic conditions [4,26]. In this respect, Kanazawa and coworkers reported that trilinolein hydroperoxides were hydrolyzed in the stomach of rats when these were administered intragastrically at low doses, giving rise to linoleic acid hydroperoxides and hydroxides. When linoleic acid hydroperoxide was administered intragastrically, it was converted into secondary oxidation compounds, including hydroxyls, epoxyketones, hexanal and 9-oxononanoic acid, in a time-dependent way [27]. The fate of linoleic acid hydroperoxides was further investigated chemically and radiochemically and these were found to decompose mainly to aldehydes and epoxyketones [28].

Regarding studies on absorption and digestibility of lipid oxidation compounds, there is general agreement that hydroperoxy, hydroxy, epoxy and oxo fatty acids are easily absorbed [29]. Back in the 1990s, digestibility of the fraction of oxidized fatty acids coming from thermoxidized oils was found to be very high in rats [30]. That fraction contained all the monomeric fatty acids with one or more oxygenated functions. These results were further supported by experiments using labeled oxidized linoleic acid [31]. Later, hydroperoxylinoleic acid and hydroxylinoleic acid, obtained enzymatically from linoleic acid, were shown to be efficiently absorbed in vitro by Caco-2 intestinal cells [32]. In humans, dietary hydroxy and epoxy fatty acids incorporated in triacylglycerols were found to be well absorbed [33,34]. Similarly, short-chain α,β-unsaturated aldehydes, of great toxicological concern, have been reported to persist after digestion [35] and to be easily absorbed [36].

The nature and amounts of oxidation compounds present in foods depend greatly on temperature. Thus, at the high temperatures of frying and baking, secondary oxidation products are most abundant, mainly hydroxides, ketones and epoxides, while hydroperoxides decompose over 150 °C [37]. In contrast, during oxidation of foods at low and moderate temperatures, substantial levels of hydroperoxides may be formed and ingested. A myriad of deleterious effects have been associated with hydroperoxides, such as in the atherosclerotic process [38] and degenerative disorders [39]. However, it is essential to get to know the contribution of compounds coming from dietary oxidized lipids in such diseases and their possible modifications throughout the gastrointestinal tract to elucidate to what extent they could be absorbed in their intact forms.

The objective of this study was to analyze some of the main chemical changes occurring to model lipid oxidation compounds specifically due to simulated gastric digestion. Model compounds selected were representative of some of the most important groups of oxidation compounds in the diet. Since epoxy, keto and hydroxy compounds are formed in significant amounts during frying and baking, methyl 9,10-epoxystearate, methyl 12-oxostearate and methyl 12-hydroxystearate were selected as model compounds. As representative of hydroperoxides, major compounds formed during oil and food storage, methyl linoleate hydroperoxides were prepared and isolated. Gastric digestion was simulated using a classical in vitro model [40]. Methyl heneicosanoate (C21:0) was used as an internal standard for quantitative determination. Gas-liquid chromatograhy/flame ionization detection (GC-FID) and gas-liquid chromatography/mass spectrometry (GC-MS) were applied in all the assays, and high-performance liquid chromatography/ultraviolet detection (HPLC-UV) was applied to hydroperoxide samples only.

## 2. Materials and Methods

### 2.1. Materials and Model Lipids

Methyl heneicosanoate (C21:0), methyl linoleate and methyl 12-hydroxystearate were supplied by Nu-Check-Prep (Elysian, MN, USA). Methyl *trans*-9,10-epoxystearate and methyl 12-oxostearate were purchased from Sigma-Aldrich (Steinheim, Germany). Silica gel 60 for column chromatography (particle size = 0.063–0.200 mm), sodium sulfate and platinum (IV) oxide hydrate were acquired from Merck (Darmstadt, Germany). All other chemicals and reagents were of analytical grade and obtained from local suppliers.

### 2.2. Preparation and Isolation of Methyl Linoleate Hydroperoxides

Methyl linoleate hydroperoxides were obtained through oxidation of methyl linoleate in the dark at room temperature following our previously published procedure [41]. Specifically, 5 g of methyl linoleate were weighed in a 250-mL beaker and oxidized for 3 days. The oxidation time was estimated so as to favor hydroperoxide formation though avoiding advanced oxidation. For that purpose, aliquots were taken periodically and analyzed by thin layer chromatography (TLC) using a blend of hexane, diethyl ether and acetic acid (80:20:1, *v*/*v*/*v*) as mobile phase. The spots were revealed by iodine vapor and the formation of hydroperoxydienes was previously confirmed by UV light. Hydroperoxides were isolated by adsorption chromatography using a chromatography column filled with silica, which was conditioned by addition of 5 wt% of water. A blend of hexane and diethyl ether (90:10, *v*/*v*) was used for the elution of the non-oxidized methyl linoleate, while the methyl linoleate hydroperoxides were eluted with diethyl ether. The fraction obtained was found to be pure by TLC and confirmation of identity and purity was performed by HPLC-UV according to Morales et al. [41], which also showed trace contents of keto and hydroxy methyl linoleate. The hydroperoxides were kept under nitrogen at −32 °C until the digestion assays were performed.

### 2.3. Samples Used in the Digestion Assays

Emulsions of model lipid compounds were prepared in a simulated gastric fluid (SGF) formulated as described in the United States Pharmacopeia [42]. SGF contained 3.2% *w*/*v* pepsin in 0.03 M NaCl and 0.1 M HCl (pH 1.2). Different sample solutions of model lipid compounds in SGF were prepared and methyl C21:0 was added as an internal standard (IS) for quantitative purposes. Homogenization was performed in an Omnimixer (Sorvall, Newton, PA, USA) at 10,000 rpm for 5 min at 1-min intervals, as described elsewhere [43]. Samples used for digestion assays are described below:

- One 15 mg mL^−1^ emulsion of a blend of methyl 9,10-epoxystearate, methyl 12-oxostearate and methyl 12-hydroxystearate (5 mg mL^−1^ each) with 10 mg mL^−1^ of the IS in SGF. Samples of 1, 2, 3 and 4 mL (~16 μmol mL^−1^ of each compound) were used.
- Two methyl 9,10-epoxysteatate emulsions, i.e., 5 and 15 mg mL^−1^, both with 10 mg mL^−1^ of the IS, in SGF. Samples of 1, 2, 3 and 4 mL (16 and 46 μmol mL^−1^) were used.
- Three methyl linoleate hydroperoxides emulsions, i.e., 5, 7.5 and 10 mg mL^−1^, with 1, 1.5 and 2 mg mL^−1^ of the IS, respectively. Additional methyl hydroperoxides emulsions at the same concentrations were prepared adjusting the pH to 3.0 and 6.6 with 1 M NaHCO_3_. Samples of 4 mL (15.3, 23.0 and 30.6 μmol mL^−1^) were used.

### 2.4. In Vitro Gastric Digestion

Gastric digestion was simulated using a classical in vitro model [40]. Emulsions of lipid model compounds prepared as described in Section 2.3. were used for digestion assays immediately after preparation. Triplicate emulsions were incubated in a shaking water bath for 3 h at 37 °C/100 rpm.

### 2.5. Lipid Extraction

Lipids were thoroughly extracted with 1:1 hexane:diethyl ether after vigorous shaking in a vortex and centrifugation at 2000 rpm. Hexane and diethyl ether have been previously used for lipid extraction of oil-in-water emulsions [43]. The extraction procedure was repeated three times and the organic extracts were pooled and washed with distilled water. After filtration through anhydrous sodium sulfate, the solvent was evaporated in a rotatory evaporator under reduced pressure at room temperature and the extracted lipids were dried using a stream of nitrogen. Control samples prepared as described in Section 2.3. were extracted after preparation to determine the extraction yield, which was always over 95%.

### 2.6. Analytical Determinations

#### 2.6.1. Quantitative Determination of Methyl 9,10-Epoxystearate, Methyl 12-Oxostearate and Methyl 12-Hydroxystearate by GC-FID

Initial and digested samples were analyzed by gas-liquid chromatography using an HP 6890 Series chromatograph (Hewlett-Packard, Avondale, PA, USA) equipped with a split-splitless injector operating in the split mode with a 40:1 split ratio at 250 °C, a J&W DB-Wax fused-silica capillary column, 60 m × 0.25 mm I.D., film thickness 0.25 μm (J&W Scientific, Folsom, CA, USA) and a flame ionization detector that was used at 250 °C. The analyses were run using hydrogen as carrier gas at 1 mL min^−1^ and under isothermal conditions using 230 °C for 25 min. Injections were carried out automatically using a 6890 Series autosampler (Agilent Technologies, Karlsruhe, Germany). A volume of 1 μL of sample dissolved in diethyl ether (~5 mg mL^−1^) was analyzed. The response factors relative to the IS (C21:0) considered for quantitative purposes were 1.23, 1.22 and 1.16 for methyl *trans*-9,10-epoxystearate, methyl 12-oxostearate and methyl 12-hydroxystearate, respectively [44].

#### 2.6.2. Quantitative Determination of Methyl Linoleate Hydroperoxides by GC-FID

Hydroperoxides were quantitated after their reduction by hydrogenation, which is absolutely necessary to stabilize them for GC analysis, since rapid degradation occurs at chromatographic conditions. Initial and digested samples of hydroperoxides were dissolved in 2 mL of methanol and hydrogenated using platinum (IV) oxide as a metal catalyst (Adams’ catalyst). Hydrogenation was carried out by bubbling hydrogen at room temperature for 10 min. Finally, methanol was evaporated in a rotary evaporator. Samples were analyzed by GC-FID under the conditions stated above. Hydrogenation transformed methyl linoleate hydroperoxides into methyl oxo- and hydroxy-stearates. Formation of methyl stearate was also observed after the hydrogenation of hydroperoxides and, consequently, also included for the quantitation of hydroperoxides.

#### 2.6.3. Analysis of Short-Chain Compounds by GC-FID

Samples of digested hydroperoxides were analyzed by GC-FID, using the chromatograph described above, under the following temperature program: 90 °C (2 min), 4 °C/min to 240 °C (25 min). Samples were introduced to the column via a split injector (split ratio 1:40) at 250 °C and the flow rate of hydrogen, used as carrier gas, was 1 mL/min. The temperature of both the split injector and flame ionization detector was 250 °C.

#### 2.6.4. Analysis by GC-MS

GC-MS analyses were performed with a Fisons MD 800 double focusing mass spectrometer operating in the electron ionization mode. Electron energy was 70 eV, the multiplier voltage was 1500 V, the source temperature was set at 200 °C and the transfer line was set at 250 °C. Spectral data were acquired over a mass range of 28–600 u at a rate of 1 scan/s. Chromatographic conditions were the same as those used for GC-FID analyses. Methyl 9,10-dihydroxysterate, methyl 9-hydroxystearate, methyl 13-hydroxystearate, methyl 9-oxostearate, methyl 13-oxostearate, methyl stearate, hexanal, methyl heptanoate, methyl octanoate, deca-2,4-dienals, methyl 8-hydroxyoctanoate, methyl 8-oxooctanoate and methyl 9-oxononanoate were identified using a commercial library (https://pubchem.ncbi.nlm.nih.gov, 3 May 2020).

#### 2.6.5. Analysis of Methyl Linoleate Hydroperoxides by HPLC-UV

Analysis of intact methyl linoleate hydroperoxides was carried out by HPLC with UV detection according to Morales and coworkers [41]. The analyses were performed in a Waters 600 chromatograph (Waters, Milford, MA, USA) equipped with a Rheodyne 7725i injector with a 20-μL sample loop and a Waters 486 absorbance detector set at 234 nm (Waters, Milford, MA, USA). The separation was performed on a silica column (LiChrospher, Si 60, 5 μm) (Merck, Darmstadt, Germany) by using *n*-heptane:isopropanol (82:18, *v*/*v*) in isocratic regime at 1 mL/min. Samples were dissolved in *n*-hexane in the range of 1–50 mg mL^−1^ prior to the analysis.

#### 2.6.6. Statistical Analysis

Initial samples were analyzed in triplicate. All the experiments were performed in triplicate and the data were expressed as means ± standard deviations. One-factor ANOVA was applied using 24.0 SPSS Statistics program (SPSS Inc., Chicago, IL, USA). Tukey’s test was used for comparisons between means and significance was defined at *p* < 0.05.

## 3. Results and Discussion

### 3.1. Fate of Methyl 9,10-Epoxystearate, Methyl 12-Oxostearate and Methyl 12-Hydroxystearate

Results obtained for equimolar mixtures containing methyl 9,10-epoxystearate, methyl 12-oxostearate and methyl 12-hydroxystearate (16 μmol mL^−1^ each) tested at different volumes (1, 2, 3 and 4 mL) are listed in Table 1.

Overall, methyl 9,10-epoxystearate was substantially lost after simulated gastric digestion, in contrast with methyl 12-oxostearate and methyl 12-hydroxystearate, which remained at the starting levels. The losses found for methyl 9,10-epoxystearate ranged from 40 to 53%. The losses decreased as the sample volume was higher, being significantly different between 1 and 4 mL. This was unexpected given that concentration of compounds in SGF was the same. Nevertheless, differences in the emulsification degree achieved or kept during digestion as the volume was increased should not be disregarded. Theoretically, reaction of lipids with the acidic medium would be diminished as emulsion stability decreased due to coalescence of lipid droplets, thus decreasing the total interfacial area [45]. Additional experiments, discussed later, were undertaken to investigate further the effect of sample volume and concentration on the loss of methyl 9,10-epoxystearate during simulated gastric digestion.

**Figure 1:**
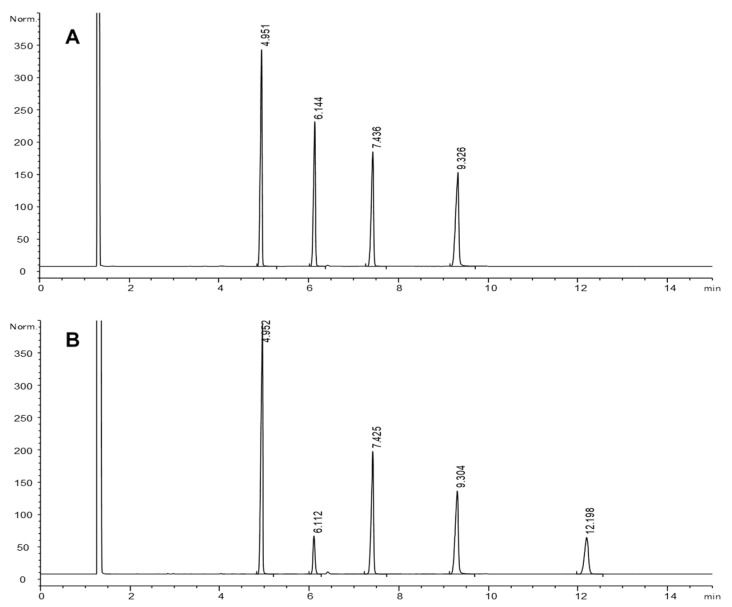
GC-FID chromatograms of a sample of the 9,10-epoxystearate, 12-oxostearate and 12-hydroxystearate equimolar mixture before (**A**) and after (**B**) simulated gastric digestion of 1 mL sample volume. Retention times (min): 4.9, methyl heneicosanoate (IS); 6.1, methyl 9,10-epoxystearate; 7.4, methyl 12-oxostearate; 9.3, methyl 12-hydroxystearate; 12.2, methyl 9,10-dihydroxystearate. Chromatographic conditions: J&W DB-Wax column; 230 °C.

Figure 1 shows representative GC-FID chromatograms illustrating the excellent resolution obtained for the mixture of methyl 9,10-epoxystearate, methyl 12-oxostearate and methyl 12-hydroxystearate. The analytical procedure here used was developed in our lab and applied to quantitate monepoxy, hydroxy and ketoacids in thermoxidized model compounds and oils. We reported that monoepoxy compounds were one of the major oxidized groups formed at frying temperatures [46,47], accounting for about 20% of the monomeric fraction of oxidized fatty acyl chains in real used frying oils at the limit of rejection.

After simulated gastric digestion, the only significant change observed in all samples was a substantial decrease of 9,10-epoxystearate and the simultaneous appearance of 9,10-dihydroxystearate, the diol expected from the opening of the epoxy ring. Figure 1 shows an example corresponding to digestion of 1 mL sample. Unfortunately, no quantitative data for the diol compound were obtained to calculate the rate of epoxystearate conversion into diol.

It is well known in industrial applications, where epoxidized fatty acid derivatives are used as plasticizers and plastic stabilizers, that diluted acid treatment and catalysis by alumina favor hydrolysis of epoxides [48]. In fact, the industrial preparation of polyols from epoxidized soybean oil using inorganic acids or methanol is of great interest [49]. As unexpected products during oxidation of conjugated linoleic acid with peroxygenase, acidic conditions used were also reported to give rise to diol products [50]. More related to conditions used in the present work, i.e., simulated gastric conditions, the scant data found in the literature only refer to cholesterol and phytosterol epoxides. Thus, Maerker et al. suggested that cholesterol epoxides could be largely hydrolyzed to render the corresponding diols [51], while Giuffrida and coworkers identified the vicinal diol derivatives obtained from cholesterol and various phytosterols after acid-catalyzed hydrolysis [52]. However, the present study provides, for the first time, quantitative data on the epoxide loss under simulated gastric conditions.

In order to investigate the effect of sample volume and concentration, two emulsions of methyl 9,10-epoxystearate (16 and 46 μmol mL^−1^) were assayed using increasing volumes. Table 2 shows the results obtained. Again, a considerably high percentage of methyl 9,10-epoxystearate was lost after digestion and this depended on both concentration and volume. The influence of the volume tested was especially noticed in the most concentrated solution, where loss percentages of 43.2 and 17.8% were found when samples of 1 mL and 4 mL were tested, respectively. As to the effect of concentration, significant differences were observed between emulsions of the same volume, probably because the stability of the emulsion decreased as the amount of the lipid phase was increased, and hence, the possibilities for reactions with the acidic medium were reduced [45]. Joint effects of concentration and volume tested were clearly reflected in the finding that methyl 9,10-epoxystearate was lost to a greater extent when the lowest concentration and lowest volume (16 μmol mL^−1^, 1 mL) was tested, while it was less modified in the sample with the highest concentration and highest volume (46 μmol mL^−1^, 4 mL).

By examining the results in Table 1 and Table 2 together, it seems that loss of methyl 9,10-epoxystearate did not depend significantly on the presence of other compounds. In fact, when comparing samples containing equal amounts of 9,10-epoxystearate and equal volumes (16 μmol mL^−1^ 9,10-epoxystearate, 1 mL and 2 mL), loss percentages were not significantly different to those found when 9,10-epoxystearate was tested alone.

Although variable results were obtained for losses of methyl 9,10-epoxystearate, overall data show the susceptibility of the epoxy ring to undergo modifications under simulated gastric conditions. Epoxide conversion to diols could be an important chemical modification occurring in the acidic environment of the stomach and, depending on the extent of the reaction, the amount of lipid compounds containing hydroxy groups ultimately absorbed could be much higher than those ingested in the diet. Nevertheless, epoxides can also be formed throughout the gastrointestinal tract, as shown in studies testing oxidized flaxseed oil in in vitro gastrointestinal digestion assays [24].

### 3.2. Changes of Methyl Linoleate Hydroperoxides

**Figure 2:**
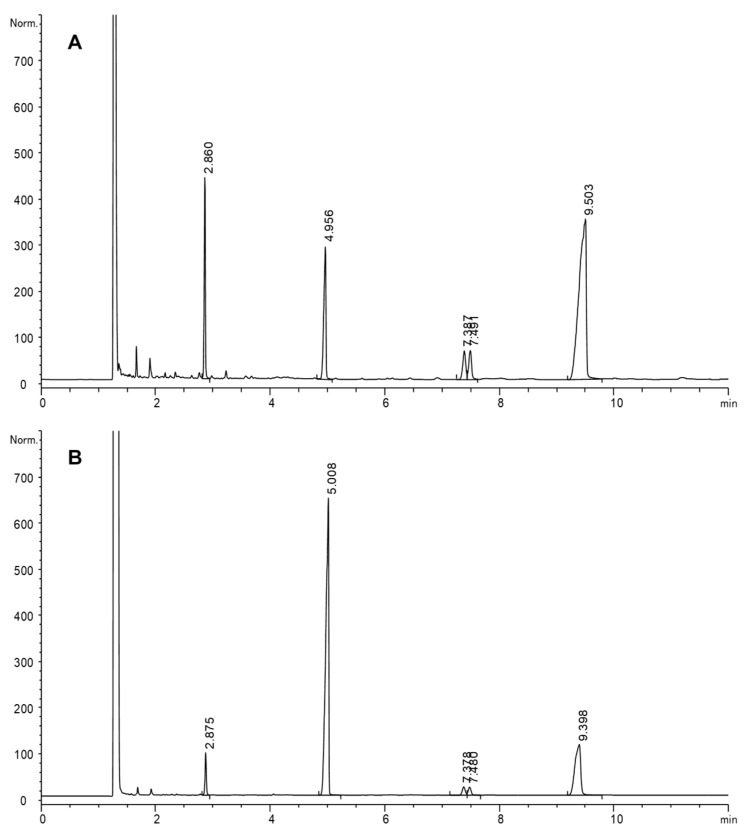
GC-FID chromatograms of a sample of methyl linoleate hydroperoxides (15.3 μmol mL^−1^) before (**A**) and after (**B**) simulated gastric digestion and both after reduction by hydrogenation. Retention times (min): 2.9, methyl stearate; 5.0, methyl heneicosanoate (IS); 7.4–7.5, methyl oxostearates, 9.4–9.5, methyl hydroxystearates. Chromatographic conditions: J&W DB-Wax column, 230 °C.

Quantitative data on hydroperoxide changes were also obtained by GC-FID. For that purpose, it was necessary to reduce methyl linoleate hydroperoxides prior to GC analysis before and after in vitro gastric digestion. Figure 2 shows an example of the application of this technique to gain insight into the changes found. Hydrogenation of original hydroperoxides (A) gave rise mainly to hydroxystearates and stearate, plus a small amount of oxostearates, as previously reported [53].

**Figure 3:**
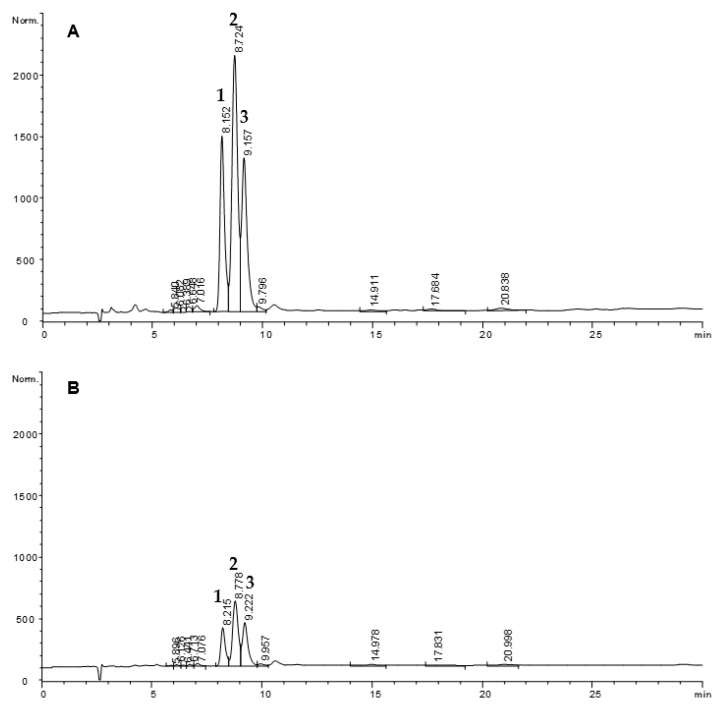
HPLC-UV (232 nm) chromatograms of methyl linoleate hydroperoxides (**1-3**) before (**A**) and after simulated gastric digestion (**B**). Chromatographic conditions: LiChrospher Si 60, 5 μm column; heptane:diethylether (88:12). Peak assignation: **1**, methyl 13-hydroperoxy-(Z)-9,(E)-11-octadecadienoate; **2**, methyl 13-hydroperoxy-(E)-9,(E)-11-octadecadienoate and methyl 9-hydroperoxy-(E)-10,(Z)-12-octadecadienoate; **3**, methyl 9-hydroperoxy-(E)-10,(E)-12-octadecadienoate [41].

Once hydroperoxides were digested and reduced (B), the quantitative data obtained by the difference between the sum of stearate, hydroxystearates and oxostearates in A and B was considered the minimum loss of hydroperoxides that took place. All peaks decreased considerably after digestion of all samples, which denotes that other secondary oxidation products not detected by this GC-FID analytical approach were formed. The profile obtained by qualitative analyses by HPLC-UV, also showed a remarkable decrease of hydroperoxides after simulated gastric conditions. An example is included in Figure 3, showing comparative chromatograms of a hydroperoxide sample (15.3 μmol mL^−1^) before and after simulated gastric digestion at pH 1.2.

As it is well-known, hydroperoxides are unstable compounds that readily decompose into a multitude of products of different molecular weight and polarity. Under the conditions used in the in vitro digestion procedure, it is likely that further reactions of hydroperoxides occurred, rendering a myriad of secondary oxidation products, including short-chain compounds and monomeric oxidation compounds. Among the latter, keto, hydroxy, epoxyhydroxy, dihydroxy, trihydroxy and other polyoxygenated compounds have been reported to be found [54]. Such polyoxygenated monomeric compounds, likely to be formed, would not elute under the GC-FID chromatographic conditions applied in the present study (Section 2.6.1), and hence, they were not detected.

Even though quantitative data were not obtained, GC-FID analysis of short-chain compounds (Section 2.6.3) showed that major volatiles were produced, namely, hexanal, methyl heptanoate, methyl octanoate, decadienals, methyl 8-hydroxyoctanoate, methyl 8-oxooctanoate and methyl 9-oxononanoate. As listed in Table 3, such volatile compounds were those mainly expected from the cleavage of the alkoxyl radicals coming from the major hydroperoxides formed in oxidized methyl linoleate (9 and 13-hydroperoxides), although others can be formed in a secondary step [54]. Thus, hexanal was the main volatile oxidation product, which comes from 13-hydroperoxide, while methyl octanoate, deca-2,4-dienal, methyl 8-hydroxyoctanoate and methyl 9-oxononanoate come from 9-hydroperoxide.

It is important to remark that other volatile compounds could have been formed, such as shorter chain aldehydes, whose proper extraction could have required solvents of higher polarity than those used; otherwise, they could not be detected under the chromatographic conditions applied. Among short chain aldehydes, α,β-unsaturated aldehydes are of particular toxicological concern, which can be formed from polyunsaturated oils under gastrointestinal conditions [20,21] and persist after digestion, [35]. Moreover, they can be released by pancreatic lipolysis from the non-volatile triacylglycerol-bound aldehydes present in dietary oils [47].

Table 4 shows the values obtained for minimum digestion loss of methyl linoleate hydroperoxides at different concentrations and pH. Availability of sufficient amounts of methyl linoleate hydroperoxides allowed further assays at different pHs. A less acidic medium at pH 3.0 was tested, considering that pH of human gastric fluid ranges from 1 to 3.5. In fact, after food ingestion, pH can increase considerably, after which it declines to acidic values [55]. Additionally, samples were tested at the lowest pH considered neutral, 6.6. The substantial effect of the acidic conditions on the stability of methyl linoleate hydroperoxides was clearly shown in the results obtained at both acidic pHs, even considering that data obtained by GC, as explained above, expressed the minimum loss observed. High percentages of methyl linoleate hydroperoxides were lost after digestion, reaching values as high as 61.7% on average for samples of lower concentration (15.3 μmol mL^−1^). No significant differences were found between the two acidic pH tested at any concentration. As to pH 6.6, only very slight losses were obtained. Clearly, under the conditions used, the acidic environment was the decisive factor for hydroperoxide changes in in vitro gastric digestion. In addition, it was found that increasing the concentration did not cause significant changes in minimum losses.

As occurs with many other variables involved in real digestion processes, great variations can be expected in the pH of the stomach, not only dependent on the gastric juice secretion but also on the buffering capacity of the ingested food [56].

The results obtained in the present work are in agreement with the few studies that have reported hydroperoxide modifications in vivo before the intestinal stage, although the authors mostly attributed these findings to the enzymatic action of glutathione (GSH)-dependent peroxidases, either in human parotid saliva [57] or in the stomach of rats [27,28]. In this latter work, Kanazawa and Ashida also showed high losses of linoleic acid hydroperoxides when these were given intragastrically to rats, i.e., about 70% decomposed to a variety of compounds, including aldehydes, epoxyketones and hydroxy compounds [28]. Additionally, in rats, Gorelik and coworkers found that stomach lipid hydroperoxides, as determined by the ferrous ion oxidation-xylenol orange method, substantially decreased 90 min after a meal of red turkey meat cutlets [58].

Regarding in vitro gastrointestinal studies, an interesting paper has been published, reporting for the first time the generation of hydroxyl-octadecadienoates from oxidized sunflower oil as determined by ^1^H NMR [23]. The authors suggested that some of the hydroperoxides originally present in the oxidized oil were reduced to hydroxyl-derivatives. Later, these results were further supported in another in vitro study using slightly oxidized soybean oil [25]. The authors found changes in the oxidation profile that were considered to be mainly due to the transformation of the initially present hydroperoxides, whose concentration diminished in the digested samples to give hydroxyl-dienes, epoxides and aldehydes. The results obtained in the present study, using isolated hydroperoxides and focused on simulated gastric conditions, provide additional information, since they clearly support that the acidic environment of the stomach is the key factor involved in hydroperoxides modifications. Notwithstanding, the formation of hydroperoxides from unoxidized oils to a variable extent has also been reported in in vitro gastrointestinal studies [4,5,6,7,8,11,12,13,14,16,25].

## 4. Conclusions

The most important conclusion obtained from the data provided in the present study is the great instability of epoxides and especially hydroperoxides under acidic conditions simulating gastric digestion. In contrast, no significant changes were found for hydroxides and ketones. Certainly, changes of lipid oxidation compounds present in foods during digestion have to necessarily be much more complex than those of model or pure compounds. Therefore, much research remains to be done to help elucidate the possible modifications of dietary lipid oxidation compounds under gastrointestinal conditions and the influence of the numerous variables involved in real food digestion processes in order to determine the structures and levels of those compounds bioavailable for intestinal absorption and, hence, for metabolic effects.

## Figures and Tables

**Table 1:** Quantitation of methyl 9,10-epoxystearate, methyl 12-oxostearate and methyl 12-hydroxystearate in an equimolar mixture, before and after simulated gastric digestion.

Sample Volume	Compound	Initial Concentration	Final Concentration	Loss % after Digestion
(mL)		(μmol mL^−1^)	(μmol mL^−1^)	
	9,10-epoxystearate	16.0 ± 0.32 c	7.52 ± 0.73 a	52.7 ± 4.6 B
1	12-oxostearate	15.95 ± 0.49 c	16.11 ± 0.21 c	
	12-hydroxystearate	15.74 ± 0.73 c	15.92 ± 0.40 c	
	9,10-epoxystearate	16.0 ± 0.32 c	7.79 ± 0.65 ab	51.3 ± 4.0 AB
2	12-oxostearate	15.95 ± 0.49 c	15.80 ± 0.37 c	
	12-hydroxystearate	15.74 ± 0.73 c	15.45 ± 1.03 c	
	9,10-epoxystearate	16.0 ± 0.32 c	8.89 ± 0.43 b	44.4 ± 2.7 A
3	12-oxostearate	15.95 ± 0.49 c	15.88 ± 0.73 c	
	12-hydroxystearate	15.74 ± 0.73 c	16.13 ± 0.39 c	
	9,10-epoxystearate	16.0 ± 0.32 c	9.55 ± 0.40 b	40.3 ± 2.5 A
4	12-oxostearate	15.95 ± 0.49 c	16.15 ± 0.58 c	
	12-hydroxystearate	15.74 ± 0.73 c	15.51 ± 1.13 c	

Results are expressed as means ± SD (*n* = 3). Different letters indicate significant differences according to Tukey´s test at *p* < 0.05. Different lowercase letters in concentration values indicate significant differences. Different uppercase letters in loss percentage calculations indicate significant differences. Loss percentages were not calculated if significant differences were not found between initial and final concentrations.

**Table 2:** Quantitation of methyl 9,10-epoxystearate before and after simulated gastric digestion.

Initial [9,10-Epoxystearate]	Sample Volume	Final [9,10-Epoxystearate]	Loss % after Digestion
(μmol mL^−1^)	(mL)	(μmol mL^−1^)	
15.79 ± 0.12 c	1	6.50 ± 1.07 a	58.8 ± 6.8 D
	2	7.20 ± 0.80 ab	54.4 ± 5.0 CD
	3	8.00 ± 0.48 ab	49.3 ± 3.0 CD
	4	8.97 ± 0.60 b	43.2 ± 3.8 BC
46.03 ± 0.94 c	1	26.16 ± 2.24 a	43.2 ± 4.9 BC
	2	30.53 ± 1.48 a	33.7 ± 3.2 B
	3	35.06 ± 0.81 b	23.8 ± 1.8 A
	4	37.82 ± 1.96 b	17.8 ± 4.3 A

Results are expressed as means ± SD (*n* = 3). Different letters indicate significant differences according to Tukey’s test at *p* < 0.05. Different lowercase letters in concentration values indicate significant differences between samples of the same initial concentrations.

**Table 3:** Major volatiles found by GC-MS after simulated gastric digestion of methyl linoleate hydroperoxides (9-OOH and 13-OOH).

Compounds	Formation Source
Hexanal	13-OOH cleavage
Methyl heptanoate	Further reactions of compounds formed from 13-OOH
Methyl octanoate	9-OOH cleavage
Deca-2,4-dienals	9-OOH cleavage
Methyl 8-hydroxyoctanoate	9-OOH cleavage
Methyl 8-oxooctanoate	Further reactions of compounds formed from 13-OOH
Methyl 9-oxononanoate	9-OOH cleavage

Formation source according to [54].

**Table 4:** Quantitation of methyl linoleate hydroperoxides before and after simulated gastric digestion at different pHs.

Initial [Methyl Linoleate Hydroperoxides]	pH	Final [Methyl Linoleate Hydroperoxides]	Minimum Loss %
(μmol mL^−1^)		(μmol mL^−1^)	after Digestion
15.30 ± 0.10 c	1.2	5.87 ± 3.29 a	61.7 ± 21.5 BC
	3.0	6.75 ± 1.80 a	55.9 ± 11.8 BC
	6.6	14.76 ± 0.25 b	3.5 ± 1.7 A
22.97 ± 0.15 b	1.2	7.98 ± 2.04 a	65.3 ± 8.9 C
	3.0	9.57 ± 1.49 a	58.3 ± 6.5 BC
	6.6	21.50 ± 0.70 b	6.4 ± 3.1 A
30.58 ± 0.25 c	1.2	14.21 ± 1.71 a	53.5 ± 5.6 BC
	3.0	17.63 ± 2.29 a	42.3 ± 7.5 B
	6.6	28.76 ± 0.80 b	6.0 ± 2.7 A

Results are expressed as means ± SD (*n* = 3). Different letters indicate significant differences according to Tukey´s test at *p* < 0.05. Different lowercase letters in concentration values indicate significant differences between samples of the same initial concentrations. Different uppercase letters in minimum loss percentage calculations indicate significant differences.

## Data Availability

Not applicable.
